# Mapping and Evaluating the Urbanization Process in Northeast China Using DMSP/OLS Nighttime Light Data

**DOI:** 10.3390/s140203207

**Published:** 2014-02-18

**Authors:** Kunpeng Yi, Hiroshi Tani, Qiang Li, Jiquan Zhang, Meng Guo, Yulong Bao, Xiufeng Wang, Jing Li

**Affiliations:** 1 Graduate School of Agriculture, Hokkaido University, Hokkaido 060-8589, Japan; 2 Research Faculty of Agriculture, Hokkaido University, Hokkaido 060-8589, Japan; E-Mails: tani@env.agr.hokudai.ac.jp (H.T.); wang@env.agr.hokudai.ac.jp (X.W.); 3 School of Urban and Environmental Science, Northeast Normal University, Changchun 130024, China; E-Mails: liq672@nenu.edu.cn (Q.L.); guom521@nenu.edu.cn (M.G.); 4 School of Environmental Sciences, Northeast Normal University, Changchun 130117, China; E-Mail: zhangjq022@nenu.edu.cn; 5 College of Geographical Science, Inner Mongolia Normal University, Hohhot 010022, China; E-Mail: baoyulong@imnu.edu.cn; 6 Northeast Institute of Geography and Agricultural Ecology, CAS, Changchun 130102, China; E-Mail: lijingsara@neigae.ac.cn

**Keywords:** Urban light index ULI, urban light space, urbanization, DMSP/OLS, Northeast China, unit circle model

## Abstract

In this paper, an Urban Light Index (ULI) is constructed to facilitate analysis and quantitative evaluation of the process of urbanization and expansion rate by using DMSP/OLS Nighttime Light Data during the years from 1992 to 2010. A unit circle urbanization evaluation model is established to perform a comprehensive analysis of the urbanization process of 34 prefecture-level cities in Northeast China. Furthermore, the concept of urban light space is put forward. In this study, urban light space is divided into four types: the core urban area, the transition zone between urban and suburban areas, suburban area and fluorescent space. Proceeding from the temporal and spatial variation of the four types of light space, the pattern of morphologic change and space-time evolution of the four principal cities in Northeast China (Harbin, Changchun, Shenyang, Dalian) is analyzed and given particular attention. Through a correlation analysis between ULI and the traditional urbanization indexes (urban population, proportion of the secondary and tertiary industries in the regional GDP and the built-up area), the advantages and disadvantages as well as the feasibility of using the ULI in the study of urbanization are evaluated. The research results show that ULI has a strong correlation with urban built-up area (R^2^ = 0.8277). The morphologic change and history of the evolving urban light space can truly reflect the characteristics of urban sprawl. The results also indicate that DMSP/OLS Nighttime Light Data is applicable for extracting urban space information and has strong potential to urbanization research.

## 1. Introduction

Urbanization is a spatial and demographic process and refers to the increased importance of towns and cities as a concentration of the population within a particular economy and society [1]. In China, urbanization is characterized by a large migration of rural populations to urban areas, which might result in the constant expansion of these urban areas. The constant construction in the new city or economic development zones are currently the mainstream sources of urban growth in China. China has undergone the highest rates of landscape changes in the World due to its changing demographics and land use practices over the last few decades [2]. By the end of 2012, the mainland of the People's Republic of China had a total urban population of 712 million or 52.6% of the total population, rising from 26% in 1990 [3]. These data indicate that cities in China have become the actual driving force for the rise of China. As the economy is developing by leaps and bounds, urban space is also expanding rapidly. Therefore, urbanization is not only of interest as a research field in economics but also falls within the scope of geography as it examines the spatial expansion of built-up areas and the morphologic characteristics of the patterns of urban area evolution.

There are presently three main types of studies on urbanization: (1) qualitative studies [4–8], which mainly study the dynamic mechanisms, regional urbanization characteristics and problems brought by urbanization (e.g., disorderly urban expansion; increased impervious surfaces in the urban areas, profound changes in the land-use type; insufficient fresh water resources, large amounts of greenhouse gas emissions and urban heat island effects) from the perspective of the population, economy and ecologic environment; (2) quantitative studies based on statistical data [9,10], in which a quantitative evaluation is conducted on regional urbanization by quantifying a series of urbanization indicators (increase of the urban population, the proportion of secondary and tertiary industries in the regional GDP and the area percentage of the built-up areas) to establish quantitative urbanization evaluation models (e.g., the urbanization rate); (3) quantitative studies based on remote sensing data [11–14], in which medium and high spatial resolution remote sensing images (e.g., Landsat TM/ETM+, SPOT HRV) are used for substantial cities. The images are employed to classify the urban landscape to study the urbanization scale and the ecologic and environmental problems produced in the process of urbanization from the perspectives of urban expansion, changes in land utilization and the evolution of urban ecologic landscape patterns. However, the qualitative evaluations cannot provide practical and effective theoretical support for urban planning department and urban decision-makers. Although the urbanization evaluation methods based on statistical data increase the scientificity of the studies on urbanization, the statistical data lacks spatial characteristics, which confine this method to the field of demography and regional economics. Thus, the crucial spatial characteristics of urbanization cannot be expounded and proved effectively. Therefore, the study of urbanization is in urgent need of precise data with spatial and temporal continuity acting as support.

Remote sensing satellite images can track the evolution of urban development in time and space. This spatial and temporal characteristic of remote sensing data compensates well for the defects of existing urbanization studies, making such data the best choice to break out of this deadlock on urbanization research [15]. Previous studies of urbanization at the regional level mainly used high resolution spatial remote sensing images, e.g., Landsat TM/ETM+, SPOT HRV, Ikonos and Worldview. However, high and medium resolution datasets are often less popular for global and national level studies. Firstly, using these datasets for national level studies involves high costs for acquiring the datasets. Secondly, these images record too much spectral information detail of urban surfaces without any screening, so massive manual handling and computer processing, time and labour are required for processing and interpreting the images [16,17]. Therefore, it is imperative to develop new approaches to timely and accurately map urban dynamics on regional and global scales with coarse spatial resolution images.

The Operational Line scan System (OLS) sensor carried by the Defense Meteorological Satellite Program (DMSP) has provided a new data approach for the study of urbanization at a large scale [18–20]. The scanning in DMSP/OLS is different from that of LANDSAT TM, SPOT HRV and NOAA AVHRR sensors, which use the reflection and radiation of surface features against the sunlight. DMSP/OLS datasets are used to map aggregate measures of urban areas such as total area extent, their ability to characterize inter-urban variation is limited due to saturation of the data values, especially in urban cores [21,22]. The purposes of this paper are the following: (1) DMSP/OLS Nighttime Light Data during 1992–2010 are utilized to extract an ULI so that analysis and quantitative evaluation of the speed and process of urbanization can be conducted; (2) through correlation analysis between ULI and the traditional urbanization indicators (urban population, proportion of the secondary and tertiary industries in the regional GDP and area of built-up area), the advantages and disadvantages as well as the feasibility of using an ULI in the study of urbanization are evaluated [23]; the unit circle model is established to make a comprehensive analysis of the urbanization process of 34 prefecture-level cities in Northeast China during the past 20 years (1992–2010). This paper is organized as follows: Section 2 describes the data and methods; Section 3 presents the results of this study; in Section 4, variation in Urban Light Indices (ULI) and urban spaces were discussed as well as the relationship between and traditional urbanization indicators; Section 5 presents conclusions and the limitations of this study.

## 2. Data and Methods

### 2.1. Study Area

**Figure 1.**
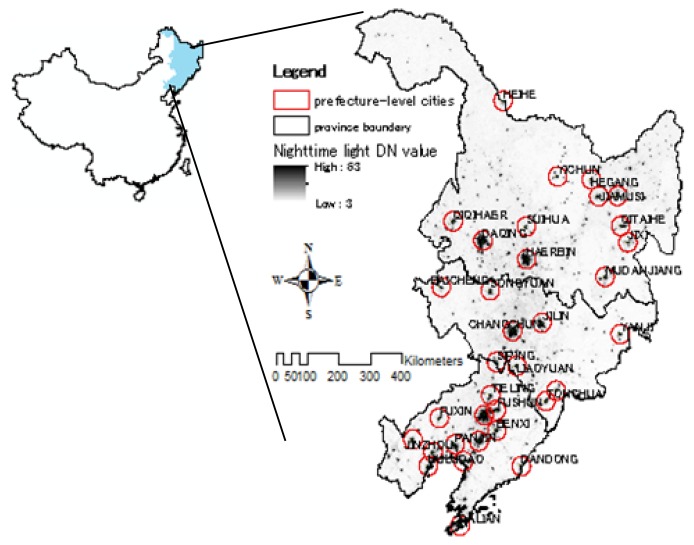
Northeast China is a geographical region of China, consisting of the three provinces of Liaoning, Jilin and Heilongjiang. There are four sub-provincial cities (Harbin, Changchun, Shenyang, Dalian) and 34 prefecture-level cities in the study region.

In this study, we focus on the Northeast region of China excluding the eastern region of Inner Mongolia (Figure 1). Northeast China contains 89 established cities with a total population of 120 million and an urban population of 31.66 million in 2010. The urbanization development is mainly thanks to its well-established railway logistics network, abundant resources and the advantages of location. After several years of development, Northeast China has become a zone of large cities located along the Harbin-Dalian railway axis. It is also a resource cities group. Meanwhile, newly emerging tourist trade cities and port cities neighboring the border and coastal areas have gradually developed too in Northeast China.

### 2.2. Data

#### 2.2.1. DMSP/OLS Night Light Data

The Defense Meteorological Satellite Program (DMSP) has an Operational Line-scan System (OLS), which is a new data source for extracting the dynamics of urban expansion at a large spatial scale [24]. The OLS sensor was placed on the DMSP Block 5D-1 satellite F-1 in September, 1976. There are two channels in the OLS sensor: (1) a visible and near-infrared channel (VNIR, 0.4–1.0 μm, 6-bit spectral resolution); (2) a thermal infrared channel (TIR, 10–13 μm, 8 bit spectral resolution). The OLS is an oscillating scan radiometer which generates images with a swath width of 3,000 km and the spatial resolution of full-resolution data is 0.56 km [25,26]. The satellite completes 14 orbits a day, and each OLS sensor can obtain all-day images covering the globe. The whole satellite system can provide observed data of the globe in four time periods: dawn, daytime, dusk and night.

In this study, we assess the urban development in Northeast China using the Version 4 global DMSP/OLS Nighttime Lights series data products that consist of 131 datasets from the six satellites in the DMSP system: F10 (1992–1994), F12 (1994–1999), F14 (1997–2003), F15 (2000–2007), F16 (2004–2009) and F18 (2010-Present). They can be obtained from National Geophysical 130 Data Center (NGDC) website (http://www.ngdc.noaa.gov/dmsp/downloadV4composites.html).

#### 2.2.2. Statistical Data

The study period (1992–2010) was divided into three periods (1992–1998; 1998–2004; 2004–2010) with an interval of 6 years. F12 1998 and F15 2004 were selected due to higher coefficients than the other alternative F14 1998 and F15 2004 (Table 1). Socio-economic indicators such as urban population, proportion of secondary and tertiary industries in the regional GDP and area of built-up area are from the China Urban Statistical Yearbook (1993, 1999, 2005, 2011) [27–30].

### 2.3. Methods

#### 2.3.1. Unit Circle Model

**Figure 2.**
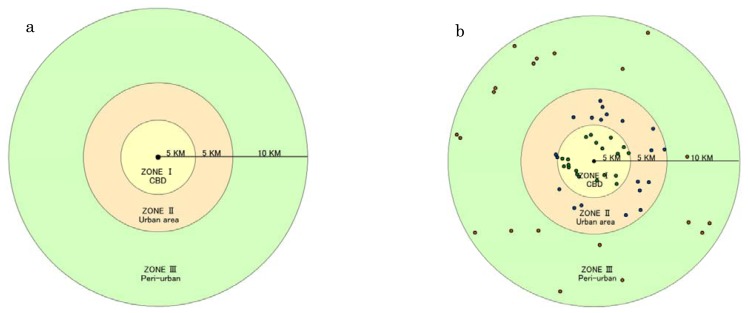
Unit circle model (**a**) and sample points (**b**).

Many urbanization literatures focus on the administrative scale in China. However, there are big variations of cities in China either in administrative spatial extent or administrative population due to some geographical, historical and political reasons. For example, HulunBuir (located in Inner Mongolia) with the administrative area of 2.6 × 10^5^ km^2^ represents the largest extent city in China, while, the administrative area in Tongling city (located in Anhui Province) is only 1,113 km^2^. Although both are prefecture-level cities, HulunBuir is 233 times larger than Tongling in administrative area. Similarly, the population of Fuyang (located in Anhui Province) is 13 million compared with the population of only 230,000 in Jiayuguan City (located in Gansu Province). These are only two notable cases, but regional variations are extremely ubiquitous in China. Therefore, it is too hard to compare the urbanization level among various cities based on administrative area due to these regional variations and we must build a ‘universal ruler’. To address this problem, a unit circle model is established in this paper, which is used to make a comparison about the scales, expansion rates of the built-up areas and the urbanization processes of different cities under the same standard. Most of the urban main train stations were constructed at city centers, and to some extent, many cities shape their spatial patterns surrounding the main train station in China. In the early stages of urbanization in Northeast China the Central Business District (CBD) is always adjacent to the main railway station due to large consumption demands of passengers, convenient transportation and information. Therefore, in this study, urban center was defined as the location of main train station of each city. With each urban center (the location of main train station) as the center, we buffered three concentric circles with a radius of 5, 10 and 30 km, respectively. Thus, instead of using the urban administrative area each city was regarded as a 20 km circle region, which can cover most cities. This circle was divided into three zonal areas from inner to outer: CBD (Central Business District) or core urban; urban area and peri-urban area (Figure 2).

#### 2.3.2. Intercalibration of the Nighttime Light Annual Composites

Recently, there has been a renewed interest in using nighttime images of Earth that show visible light emissions, providing a dramatic picture of urbanization through long term city lights monitoring [31]. Although NTL data do not measure land cover directly and many non-urban places are lit at night, including agricultural fields and fishing vessels, it has been shown to be strongly correlated with population density [32]. However, the DMSP-OLS NTL time series dataset cannot be directly used to study urbanization due to the absence of on-board calibration in the OLS [25,33]. For each year, NTL data acquired by different satellites has no strict intercalibration. The lack of continuity and comparability means that these data cannot be directly used to extract the dynamics of global and regional urban expansion. In order to reduce these discrepancies and impart comparability to the NTL dataset it is essential that the data should be intercalibrated first.

In order to improve the consistency and comparability of the NTL time series dataset in China from 1992 to 2010, we followed an empirical procedure which is the so-called second order regression model proposed by Elvidge in 2009 [34]. We created 20 random points within each zonal area of the unit circle, so we got 60 random points for each city (Figure 2). The 34 total prefecture-level cities in Northeastern China were treated in the same way. Then, all the random points were taken as the samples to establish the empirical relationship for intercalibration. These sample cities and towns were extracted from each zonal area (CBD, urban area and peri-urban). In reviewing the NTL time series dataset, it was found that the data from the satellite year F162007 captured with maximum number of lit pixels in the region of China (Table 1). Therefore, F16 2007 was used as the reference and the data from all other satellite years were adjusted to match the F162007 data range. The highly conformal Digital Number (DN) values along the time series for the pixels in the calibration area will ensure high R^2^ values for the empirical equation. Intercalibration was conducted by using second order polynomial regression (Equation 1), with the dependent variable as the reference image and the independent variable as images to be calibrated (Table 1). The model parameters a, b and c were estimated with ordinary least square regression for individual NTL image. The regression equation was then applied to individual DMSP-OLS NTL image to calculate the adjusted DN values [35]:

(1) $${\text{DN}}_{\text{calibrated}}=\text{a}\times {\text{DN}}^{2}+b\times DN+c$$

#### 2.3.3. Urban Light Index (ULI)

Each grid of Version 4 DMSP/OLS nighttime light data products has a radian of 30 s, and the coverage area is −180°∼180°E, −65°∼75°N. The range of Digital Number (DN) values of each pixel is 0–63. The DN value directly indicates the intensity of the area's light. The DN value of the pixels in non-lit areas is zero. A pixel with a DN value of 63 is a saturation pixel and most of saturation is found in the core urban area [36–38]. The nighttime light grid data images record the light intensity (indicated by the DN value) and spatial extent information of cities (indicated by the lit pixel counts). The lit areas representing urban area may “grow” slightly around their light source due to “blooming” and noisy data associated with the urban fringe areas. This has the effect of enlarging small towns and expanding the boundaries of large cities [39]. Using all the data in the NGDC city-lights data with DN > 7 were believed to represent urban areas [40]. By comparing many cities and rural areas, we consider DN > 10 as the city lights in this work. Therefore, these two attributes are adopted to construct area light indices. The calculation formula is:

(2) $$\text{ULI}=100\sum_{i=10}^{\max DN}\frac{{\text{DN}}_{i}}{\max\text{DN}}\times \frac{C_{i}}{\text{sumC}}$$

where ULI is the nighttime urban light index and DN_i_ is the calibrated DN value by Equation (1). C_i_ and sum C are the count of DN_i_ and all lit pixels in a unit circle area, respectively. Here, $\frac{{\text{DN}}_{\text{i}}}{\text{maxDN}}$ indicates the brightness which reflects the light intensity of each urban area, while $\frac{{\text{C}}_{\text{i}}}{\text{sumC}}$ reflects the weight of DN_i_ in unit circle extent. According to Equation (2), the Urban Light Indices (ULI) of the 34 prefecture-level cities were calculated within the unit circle extent.

## 3. Results

### 3.1. Urban Light Index (ULI)

As mentioned above, it is too hard to compare the urbanization level among various cities based on administrative area due to great differences either in spatial extent or population. In order to address this problem, a unit circle model is established in this paper, which is used to make a comparison about the scales, expansion rate of the built-up areas and the urbanization processes of different cities under the same standard. With each urban center as the center we buffered a circle area with a radius of 20 km in order to cover the built-up areas of each city.

**Figure 3.**
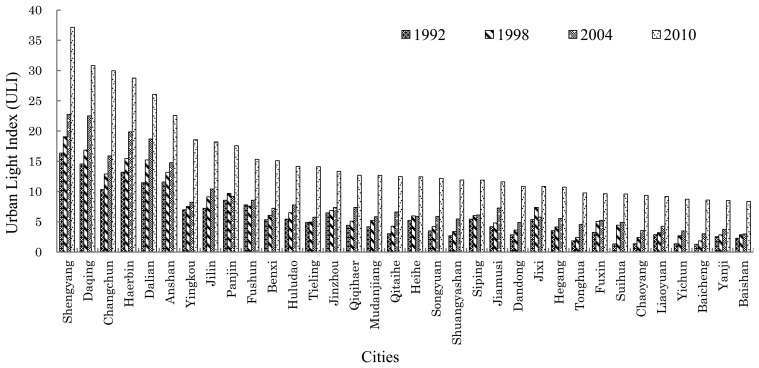
ULI dynamics in the Northeast region of China.

Figure 3 shows the ULI results of the 34 prefecture-level cities in Northeastern China. As the most developed regional central cities in China, Harbin, Changchun, Shenyang and Dalian are also centers of economic and social development in the northeastern region. The evolution in population, economy and social activities in these four cities represents the urbanization level and progress in Northeast China. The systems of the northeastern cities are mainly divided into the following three levels: the first level, Harbin and Dalian traffic axis zone, integrates developed railways, highways, airports, ports and information network systems. In past twenty years, this axis city zone has further developed and become the backbone of urban and economic development in Northeastern China. In the second level are the mineral resource-based industrial cities, including Daqing, Shuang yashan, Qitaihe, Jilin, Siping, Fushun, Anshan, Benxi and Tieling. In the third level, some newly emerging tourist trade cities and port cities appear taking advantage of their proximity to the coastal area and borders. These cities mainly include Heihe, Dandong, Yingkou, Jinzhou and Huludao.

### 3.2. Urban Light Space

From the perspective of geographical space, two expansion forms in the built-up area, vertical continuation and horizontal development, happen in the urbanization process. The former enhances the intensity of urban light; while the later enlarges the extent of urban light. In this paper, the so-called Urban Light Space is the space illuminated by urban lights at night, and is not the actual urban area; rather, it is the spatial range of the urban light area observed by satellite at night. In accordance with different distributions in urban light intensity, the Urban Light Space is classified into four types: core urban area (DN 57–63), urban and suburban transition zone (DN 45–57), suburban area (DN 26–45) and urban fluorescence space (DN 11–25). Table2 shows the transformations and expansion ratio of urban light space types from 1992 to 2010.

### 3.3. The ULI and Traditional Urbanization Indicators

The urbanization level has become an important criterion for measuring the economic activities in a country or district and the level of social progress. Urbanization mainly includes three dynamic variations, *i.e.*, population, economy and urban building areas. Three important urbanization indicators, population (proportion of non-agricultural population to total population), economy (proportion of output value of secondary and tertiary industries) and urban building area (built-up area), are selected in this paper to compare the variation in ULI. For statistical and remote sensing data, four sub-provincial cities, Harbin, Changchun, Shenyang and Dalian, were selected to analyze the characteristics of temporal and spatial urbanization process from 1992 to 2010 (Table 3).

## 4. Discussion

### 4.1. Spatial and Temporal Pattern Variation in Urban Light Indices (ULI)

**Figure 4.**
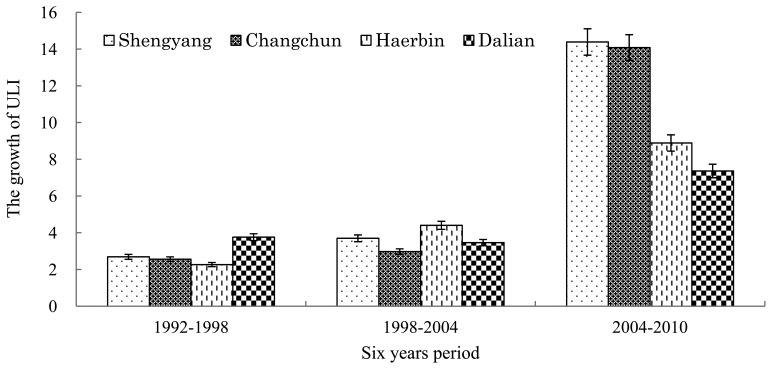
ULI growth of four sub-provincial cities.

During the past twenty years, the ULI in these four central cities underwent a fluctuating increase (Figure 4). The differences between the ULI increases of Harbin, Changchun and Shenyang are small, while that of Dalian increased greatly during the period from 1992 to 1998. That is because unprecedented and large-scale urban construction projects were completed in Dalian in the early and middle 1990s. After 20 years of development, a new pattern of urban space has formed around the bay of Dalian, including the old city, and two new districts, Jinzhou and Jingang.

**Figure 5.**
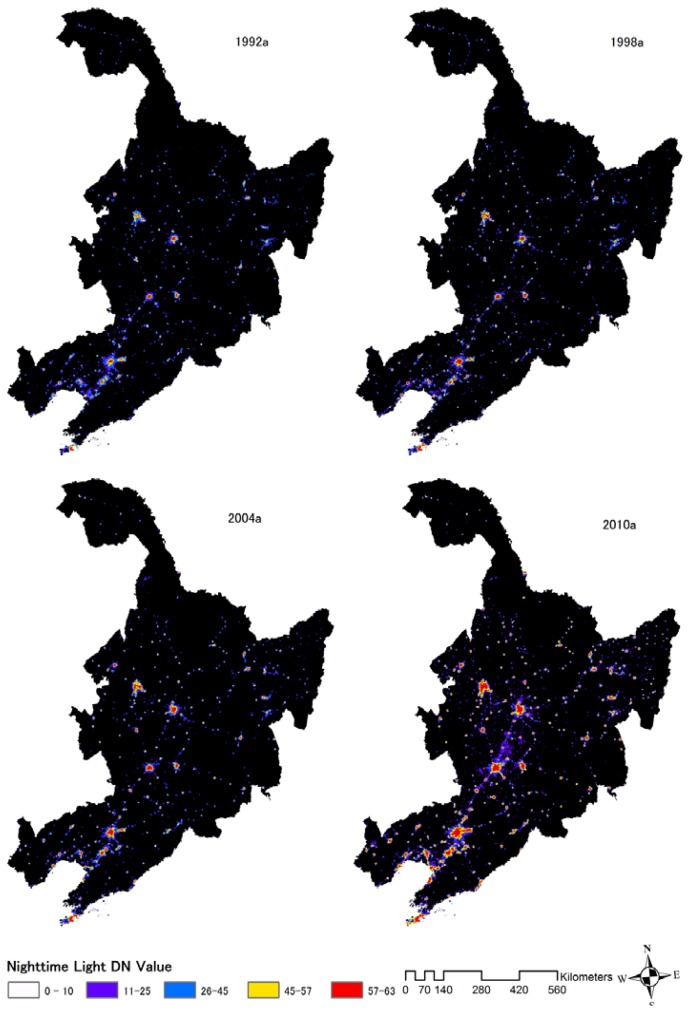
Urban nighttime light dynamics in the Northeast of China from 1992 to 2010.

The formation process of this pattern is based on urban population, economic activities and the dynamic variation of urban sizes. Meanwhile, urban nighttime light satellite images have objectively recorded the development track of temporal-spatial pattern variation of these cities. The urbanization level is higher than the average national level, and the dynamic mechanism is typical and of distinct characteristics during the urbanization process. Thirty four prefecture-level cities in Northeast China were selected to analyze the development of large cities in this region during the past twenty years (1992–2010). Four sub-provincial cities, Harbin, Changchun, Shenyang and Dalian are selected to analyze the characteristics of the temporal and spatial urbanization process on aspects of spatial expansion of urban lights and dynamic variation in urbanization indices (Figure 5).

**Figure 6.**
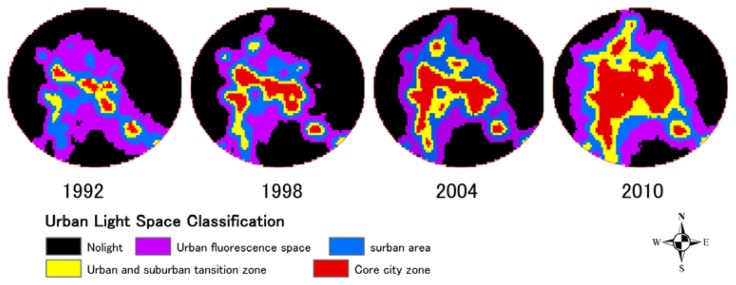
The trajectories of multicenter city in the urbanization process (such as Daqing city).

The ULI of the 34 prefecture-level cities in Northeast China can reflect the development levels of these cities. In the ULI ranking of the 34 prefecture-level cities, it is no surprise that the four sub-provincial cities, Shenyang, Changchun, Harbin and Dalian, are on top of the ranking. Meanwhile, it can be noticed that the ULI of Daqing and Anshan unexpectedly rank in the second and the fifth place, respectively. That is because Daqing is a city growing up from the first oil field in China and there are approximately 3,500 (2010 data) oil wells working throughout the day and night in each district of the city. In addition, many petrochemical plants of this city are also lit, which makes ULI in Daqing much higher than in other cities. However, it can be found from the nighttime light images that the ULI range in Daqing is remarkable, as there are many discontinuous areas with low light intensity values, and the urban space structure has discrete multicenter characteristics (Figure 6). Similarly, Anshan is also a newly emerging industrial urban like Daqing. High-intensity lights from factories and workshops of the Anshan Iron and Steel Group and rock quarries around the urban area also lit at night, therefore, the light index of Anshan is listed in fifth place among 34 the northeastern cities.

These results suggest that urbanization level will be overestimated in industrial cities by using ULI. During the years from 1992 to 2010, the ULI of nearly all of the 34 prefecture-level cities in Northeast China had increased during different periods, especially from 2004 to 2010, when the ULI of all cities rose dramatically to varying degrees. There are two reasons: on one hand, the increase in urban light index is the result of urbanization. On the other hand, the lighting projects (also known as urban lights projects, which means lights in places with intensive population, such as malls, scenic spots and streets, to beautify the urban environment and improve the whole image of the city) have lit entire cities since the turn 21st century, with the improvement in economic level and further improvements of urban infrastructures during the period from 2004 to 2010, therefore, the ULI in large cities has risen sharply.

### 4.2. Trajectories and Scenarios of Urban Light Space Evolution

**Figure 7.**
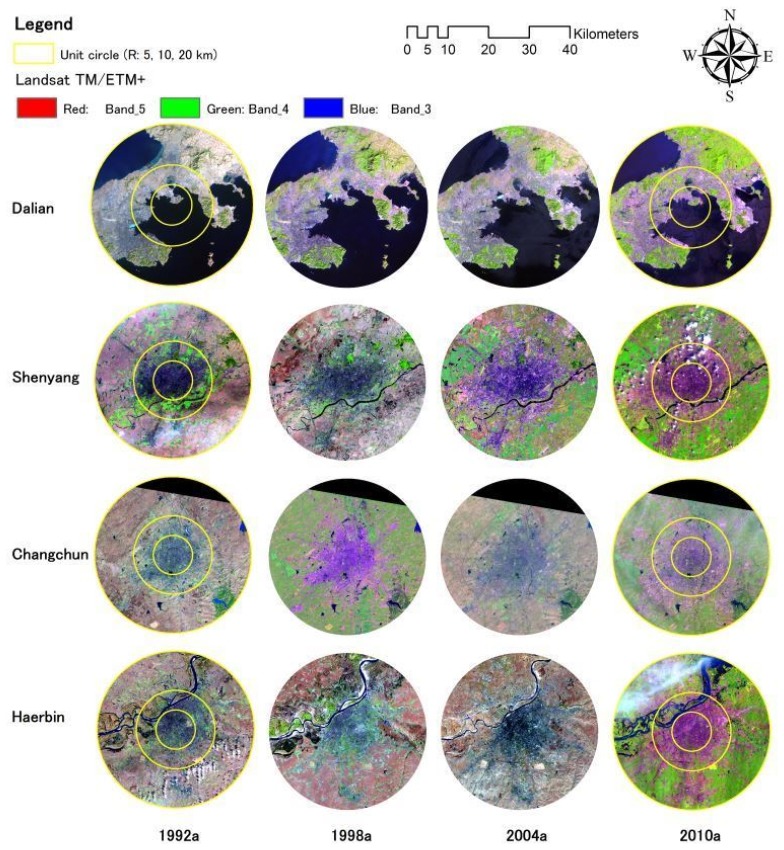
Maps of the morphology evolutions of four sub-provincial cities.

**Figure 8.**
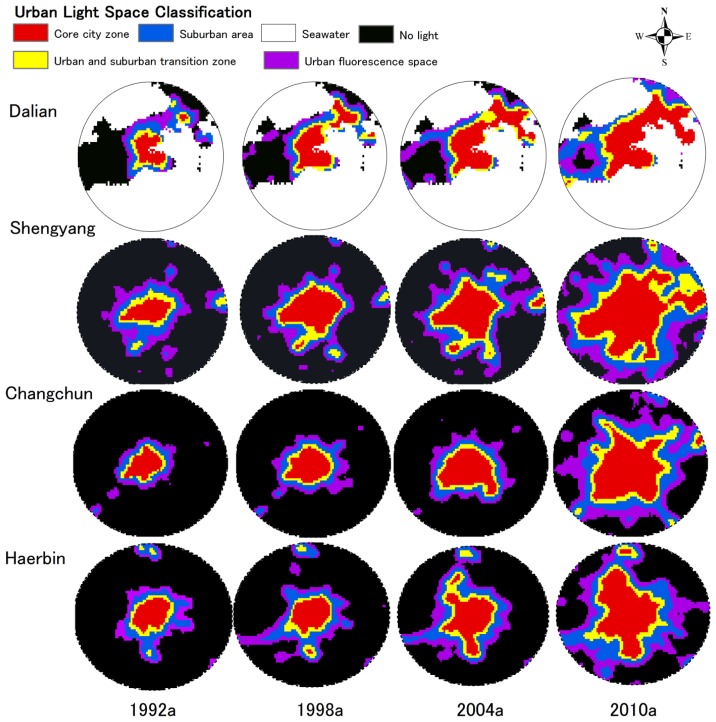
Urban light space sprawl scenarios of four sub-provincial cities from 1992 to 2010.

With the center of each city as the center of a circle, a circular region with a radius of 20 km is established in each Urban Light Space. Figure 7 shows the morphological characteristics and spatial and temporal evolution of four sub-provincial cities. At the same time, the Urban Light Spaces of these four cities in each stage show a trend of obvious expansion from 1992 to 2010 (Figure 8).

Shenyang is a core city in the northeast area, and its Urban Light Space area is also listed at the top of these four cities. The light space in the Shenyang core urban area has undergone two rapid expansion periods, from 1992 to 1998 and from 2004 to 2010. The light space in the core urban reaches 834 km^2^. The urban process has further quickened since the proposal of the Shenyang and Fushun development plan. The ecological landscape corridor, with Hun River as the axis and the new city of Shenfu (the fusion of the cities of Shenyang and Fushun) make these two cities closely related, and the effect of urban integration of “one urban, two districts and an area” has become increasingly more obvious.

Judging from the spatial-temporal evolution of the light space, the urban spatial morphology of Dalian has developed dramatically in past twenty years along Liaodong Peninsula. The expansion of light space in the core urban area reached the peak in the 1990s, after which it gradually slowed down. However, the light space Dalian city as a whole continues to display a stable rising tendency. Dalian is located at the southernmost point of the Liaodong Peninsula. The urban space can only be expanded along the axis of the peninsula to north and developed vertically to the south due to the geographical space limitations. Large-scale urban construction and reconstruction projects were carried out in Dalian in the 1990s. After twenty years of development, a new urban space pattern connecting the old city of Dalian and the new cities of Jinzhou and Jingang has formed around Dalian Bay. Changchun City rapidly developed from 2004 to 2010. The original concentric circle surface structure form of expansion changed to a dispersed group development trend. With the transfer of the city center, Xinlong, Jingyue and Fufeng groups were formed around the old urban space. Viewed from the Urban Light Space of the evolution process diagram (Figure 8), the urban space of Harbin is farther south and north and has a flourishing central area. During the recent twenty years, the light area of the core city has increased from 154 km^2^ to 579 km^2^. The suburban transition zone is gradually expanding outwards, and the rate of suburban urbanization has further increased since the beginning of the 21st century.

Due to light scattering and diffraction effects, fluorescence spaces with large areas have formed around the cities. Generally speaking, the spatial area of urban light is much larger than the actual range of the urban areas. However, the history of the formation and evolution of Urban Light Space can faithfully reflect the objective features of urban space evolution. Meanwhile, we must admit that the lighting projects in cities will inevitably influence the Urban Light Space, which is also one reason of uncertainty in this research.

### 4.3. Relevance Analysis between the ULI and Traditional Urbanization Indicators

Urbanization is a comprehensive human activity phenomenon accompanying the process in which a large rural population migrates to the city, the city economy develops rapidly, and the urban space constantly expands in the horizontal and vertical directions. Population, economic activity and built-up are significant indicators for the regional urbanization process. Some scholars have proposed that urban population, economic activities, built-up area and Urban Light Space range are of high relevance [41–43].

**Figure 9.**
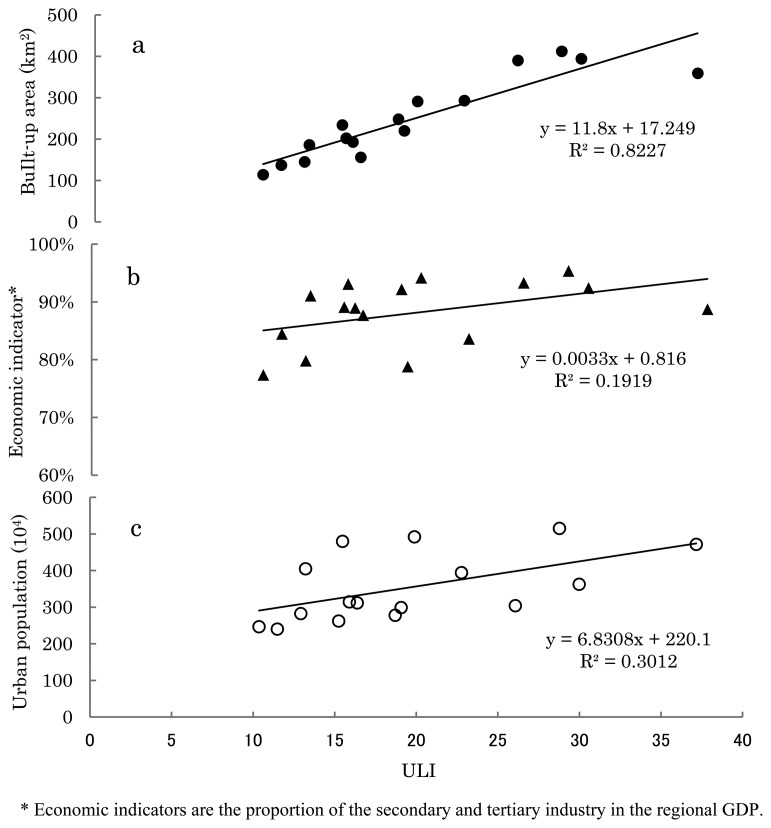
Correlation between nighttime light index and common urbanization indicators. (**a**) BuIlt-up area, (**b**) Economic indicator, (**c**) Urban population.

Various urbanization indicators of the four sub-provincial northeastern cities from 1992 to 2010 are analyzed in this paper along with urban light indices. Table 2 and Figure 8 further illustrate that ULI has a strong correlation with urban built-up area (R^2^ = 0.8277) but low correlation with an urban population and economic activities. In China, government policy plays a crucial role in the build-up areas which is the key factor of city light. In areas with a high level of economic development, both the intensity of city light and the area of Urban Light Space can be clearly observed in the remote sensing images of night lights. Due to the existence of light scattering and diffraction effects, the Urban Light Space, more often than not, exceeds the range of the real city space. It is worth noting that DMSP/OLS night light images can still accurately show the features of variation in city space structures. Moreover, the city light index and the built-up area are of high relevance for statistical data (Figure 9a).

## 5. Conclusions

The urbanization process exhibits obvious geographical spatial attributes. DMSP/OLS night light data has become a better choice for urbanization research than other daytime satellite data for the following two reasons: (1) the spatial resolution of DMSP/OLS night light data is 1 km, it is are able to cover all human residential areas in the World and are applicable for regional urbanization research [44,45]; (2) against a dark background, the night light data images remove the disturbances of redundant information on city surface landscapes in daytime images so they can sensitively capture the outline of the cities, which is beneficial for extracting city space information.

In this paper, an ULI is constructed to analyze and quantitatively evaluate the speed and process of urbanization by using DMSP/OLS Nighttime Light Data from the period from 1992 to 2010. The urban light intensity is able to reflect the agglomeration level of a city's population and economic activities, while, the variation in Urban Light Space records the trace of urban space expansion.

To compare the urbanization levels in different cities under the same standard, a unit circle urbanization evaluation model is established in this paper to comprehensively analyze the urbanization process in 34 prefecture cities in Northeast China during the recent twenty years (1992–2010). Influenced by factors such as location, history and politics, there are considerable regional differences in the administrative areas of the different cities and population sizes, with no transverse comparability. Therefore, urbanization research with the administrative area as the evaluation unit cannot objectively reflect the true development rate and evolution. To a large extent, the unit circle model removes the disadvantages of urbanization research with the administrative area as the evaluation unit. Comparatively speaking, the unit circle model is more applicable to the evaluation and research of urbanization level among cities within a region.

By comparing the relevance analysis on ULI and traditional urbanization indices (urban population, proportion of GDP in the secondary and tertiary industries and the built-up area), Urban Light Indices (ULI) are evaluated to research the advantages and disadvantages as well as the feasibility of urbanization research. The research results show that ULI, urban population, economic indicators and area of built-up areas in cities are of strong relevance. The form of Urban Light Space and the history of development can faithfully reflect the objective features of urban space evolution. There is linear relevance among the urban population, economic activity, built-up area and linearity of ULI (the correlation coefficients are 0.3, 0.2 and 0.8, respectively). The concept of Urban Light Space is put forward in this paper for the first time. The Urban Light Space is divided into core urban area, suburban transition zone, suburban area and urban fluorescence space. In accordance with different distributions in urban light intensity, the Urban Light Space is divided into core urban area (DN 57–63), suburban transition zone (DN 45–57), suburban area (DN 25–45) and urban fluorescence space (DN 15–25). Viewed from the temporal and spatial variation of these four light space types, the urban forms and temporal and spatial evolution patterns of the four northeastern cities of Harbin, Changchun, Shenyang and Dalian, are paid particular attention in this paper. The results revealed that the ULI and Urban Light Space can accurately reflect the features of temporal and spatial pattern of cities in the urbanization process. It is further illustrated that DMSP/OLS night light data is more applicable to urban space information extraction and urbanization process research.

## Figures and Tables

**Table 1.** Equations for intercalibrating the annual nighttime lights products. The adjusted digital number (DN) is created by the application of this formula. The coefficients are empirically derived by comparing images of China region with F162007.

**Satellite**	**Year**	**c**	**b**	**a**	**R**^2^	**Second-Order Regression**
**F10**	1992	1.0287	0.0895	0.0085	0.721	y = 0.0085x^2^ + 0.0895x + 1.0287
	1993	1.3181	0.0661	0.0084	0.747	y = 0.0084x^2^ + 0.0661x + 1.3181
	1994	1.2465	0.0629	0.0091	0.751	y = 0.0091x^2^ + 0.0629x + 1.2465

**F12**	1994	0.8022	0.2150	0.0085	0.795	y = 0.0085x^2^ + 0.215x + 0.8022
	1995	1.3061	0.1706	0.0090	0.808	y = 0.009x^2^ + 0.1706x + 1.3061
	1996	1.1985	0.1710	0.0087	0.808	y = 0.009x^2^ + 0.171x + 1.1985
	1997	1.1741	0.2526	0.0084	0.827	y = 0.0084x^2^ + 0.2526x + 1.1741
	1998	1.1838	0.2928	0.0081	0.849	y = 0.0081x^2^ + 0.2928x + 1.1838
	1999	0.9326	0.2928	0.0084	0.858	y = 0.0084x^2^ + 0.2985x + 0.9326

**F14**	1997	1.2930	0.0326	0.0107	0.795	y = 0.0107x^2^ + 0.0326x + 1.293
	1998	1.4549	0.0581	0.0106	0.822	y = 0.0106x^2^ + 0.0581x + 1.4549
	1999	1.4191	0.0738	0.0108	0.838	y = 0.0106x^2^ +0.0738x + 1.4191
	2000	1.3943	0.1275	0.0101	0.848	y = 0.0101x^2^ + 0.1275x + 1.3943
	2001	1.2523	0.2012	0.0097	0.872	y = 0.0097x^2^ + 0.2012x + 1.2523
	2002	0.9366	0.2967	0.0093	0.901	y = 0.0093x^2^ + 0.2967x + 0.9366
	2003	0.6364	0.4101	0.0083	0.933	y = 0.0083x^2^ + 0.4101x + 0.6364

**F15**	2000	1.4330	0.3334	0.0079	0.868	y = 0.0079x^2^ + 0.3334x + 1.433
	2001	1.3663	0.3612	0.0076	0.881	y = 0.0076x^2^ + 0.3612x +1.3663
	2002	1.0814	0.5267	0.0060	0.917	y = 0.006x^2^ + 0.5267x + 1.0814
	2003	0.9064	0.2774	0.0096	0.923	y = 0.0096x^2^ + 0.2774x + 0.9064
	2004	0.6344	0.4076	0.0083	0.946	y = 0.0083x^2^ + 0.4076x +0.6344
	2005	0.6281	0.4850	0.0071	0.955	y = 0.0071x^2^ + 0.485x + 0.6281
	2006	0.2504	0.6042	0.0057	0.979	y = 0.0057x^2^ + 0.6042x + 0.2504
	2007	0.1775	0.6308	0.0049	0.983	y =0.0049x^2^ +0.6308x + 0.1775

**F16**	2004	0.7564	0.6403	0.0050	0.936	y = 0.005x^2^ + 0.6403x + 0.7564
	2005	0.8615	0.4648	0.0073	0.963	y = 0.0073x^2^ + 0.4648x + 0.8615
	2006	0.2173	0.7822	0.0029	0.976	y = 0.0029x^2^ + 0.7822x + 0.2173
	2007	0.0000	1.0000	0.0000	1.000	y = x
	2008	−0.0870	1.0258	−0.0003	0.983	y = −0.0003x^2^ + 1.0258x − 0.0870
	2009	0.0057	1.0388	−0.0017	0.897	y = −0.0017x^2^ + 1.0388x + 0.0057

**F18**	2010	1.3743	0.1277	0.0121	0.848	y = 0.0121x^2^ + 0.1277x + 1.3743

**Table 2.** Nighttime urban light space classes and their dynamics.

**City**	**Year**	**Core Urban Area**		**Transition** **Zone**	**Suburban** **Area**	**Fluorescence** **Space**	**Light Space of Whole** **Urban**	

		**Area** **(km^2^)**	**Expansion** **Ratio (%)**	**Area** **(km^2^)**	**Area** **(km^2^)**	**Area** **(km^2^)**	**Total Area** **(km^2^)**	**Expansion** **Ratio (%)**
**Haerbin**	1992	154.56		90.39	242.19	222.18	709.5	
	1998	181.47	17.4%	105.57	284.97	328.44	901.2	27.0%
	2004	319.47	76.0%	186.3	360.87	350.52	1219.3	35.3%
	2010	579.6	81.4%	247.71	557.52	585.81	1972.8	61.8%

**Changchun**	1992	124.89		53.82	103.5	144.9	427.2	
	1998	184.92	48.1%	75.21	155.94	224.25	641.9	50.2%
	2004	283.59	53.4%	104.19	171.12	224.94	785.1	22.3%
	2010	580.98	104.9%	273.24	552	644.46	2056.1	161.9%

**Shenyang**	1992	134.55		123.51	271.86	345	875.1	
	1998	304.98	126.7%	157.32	244.95	282.9	991.8	13.3%
	2004	376.74	23.5%	224.25	371.22	382.95	1356.7	36.8%
	2010	834.21	121.4%	405.03	612.03	578.91	2433.5	79.4%

**Dalilan**	1992	112.47		81.42	184.92	144.21	523.2	
	1998	233.22	107.4%	124.89	154.56	144.21	658.5	25.9%
	2004	349.83	50.0%	132.48	145.59	152.49	781.1	18.6%
	2010	460.92	31.8%	131.1	260.82	146.97	1001.2	28.2%

**Table 3.** ULI and urbanization indicators of four cities.

**City**	**Year**	**ULI**	**Traditional Urbanization Indicators**		

			**Urban Population (10^4^)**	Economic Indicators *(%)	**Built-up Area (km^2^)**
**Shenyang**	1992	16	311.98	87.7%	156
	1998	19.07	299.16	78.8%	220
	2004	22.77	394.54	83.6%	293
	2010	37.16	471.79	88.7%	359

**Changchun**	1992	10	246.81	77.4%	114
	1998	12.92	282.69	79.8%	145
	2004	15.90	314.7	89.0%	193
	2010	29.98	362.75	92.4%	394

**Haerbin**	1992	13	405.1	91.1%	186
	1998	15.48	479.99	93.1%	202
	2004	19.89	492.34	94.2%	291
	2010	28.77	515.42	95.4%	412

**Dalilan**	1992	11	240.33	84.5%	137
	1998	15.24	262.4	89.1%	234
	2004	18.71	278.09	92.2%	248
	2010	26.07	304.26	93.3%	390

* Economic indicator is the proportion of the secondary and tertiary industry in the regional GDP.
